# Draft Genome Sequences of Robbsia andropogonis Isolated from Sorghum bicolor Exhibiting Bacterial Leaf Stripe in Australia

**DOI:** 10.1128/mra.00247-22

**Published:** 2022-09-21

**Authors:** Anthony J. Young, Henry W. G. Birt, Robert D. Hoelzle, Paul G. Dennis

**Affiliations:** a School of Agriculture and Food Sciences, The University of Queensland, Gatton, QLD, Australia; b School of Earth and Environmental Sciences, The University of Queensland, Brisbane, QLD, Australia; SIPBS, University of Strathclyde

## Abstract

Robbsia andropogonis causes leaf spots, streaks, or stripes on a wide range of commercially important crops. Here, we present the draft genome sequences of two isolates of *R. andropogonis* sourced from Sorghum bicolor displaying symptoms of bacterial leaf stripe disease in Australia.

## ANNOUNCEMENT

Robbsia andropogonis bacteria are pathogens that affect a wide range of crops around the world (1–7). Despite their global importance as phytopathogens, only three *R. andropogonis* isolate genome sequences are available currently through GenBank (8) (BioProjects PRJEB35318, PRJNA279601, and PRJNA228914), which are all sourced from the United States. Here, we present draft genome sequences for two *R. andropogonis* isolates (BRIP 72872a and BRIP 72957a) sourced from Sorghum bicolor plants displaying symptoms of bacterial leaf stripe disease in Australia.

Sorghum samples exhibiting symptoms of bacterial leaf stripe were collected from the field at Jondaryan (27.3678° S, 151.5907° E), Queensland, Australia, in March 2016. Leaf samples with bacterial ooze were surface sterilized and plated onto nutrient agar medium (25°C). Isolates were purified by successive streaking of individual colonies and then subjected to DNA extraction according to the method of Doyle and Doyle (9), which involves mechanical processing of the biomass in cetyltrimethylammonium bromide (CTAB) buffer and purification in chloroform-isoamyl alcohol. DNA was further purified using DNA spin columns from the Isolate II plant DNA extraction kit (Bioline). DNA libraries were prepared using the Nextera XT DNA library preparation kit (Illumina, Inc., CA, USA) and sequenced on the MiSeq platform with 150-bp paired reads (Illumina, Inc.) generating a total of 1 Gbp and 860 Mbp of data from 2.9 million and 3.4 million paired-end reads for BRIP 72957a (SRR15602133) and BRIP 72872a (SRR15602134), respectively. For both isolates, there were two reads per spot and an average read length of 148 bp.

Raw reads were trimmed and quality filtered with Trimmomatic v0.36 (10) using the following settings: ILLUMINACLIP: NexteraPE-PE.fa: 2:30:10; TRAILING:10; SLIDINGWINDOW:4:15; and MINLEN:75. Paired-end trimmed reads were *de novo* assembled using SPAdes v3.14.1 (11) in isolate mode. The assembled contigs within each genome sample were then checked for completeness and contamination using the CheckM v1.1.2 (12) and were assigned taxonomy using GTDB-Tk v1.3.0 and the GTDB 207 release database (13). The NCBI Prokaryotic Genome Annotation Pipeline (PGAP; v6.1) was used to identify genes within the genomes (14). Average nucleotide identity (ANI) was assessed with FastANI v1.3 (15). Default parameters were used for all software unless otherwise specified.

The BRIP 72872a and BRIP 72957a draft genomes were of similar size (6.44 and 6.43 Mbp, respectively) and composition (99.9% ANI) to each other and to the full genome of the *R. andropogonis* type strain (GCF_000970345.1; 6.20 Mbp, 99.5% ANI each). The BRIP 72872a and BRIP 72957a draft genome sequences were assembled into 344 and 305 contigs, respectively, and both had *N*_50_ scores of 216 Kbp and a GC content of 58%. According to CheckM, the draft genomes of BRIP 72872a and BRIP 72957a were 98.66% and 98.26% complete, with 2.01% and 1.07% contamination, respectively. PGAP annotated 5,745 genes, including 3 copies of the 16S rRNA gene in BRIP 72872a, and 5,717 genes and 3 copies of the 16S rRNA gene in BRIP 72957a. For reference, the *R. andropogonis* type strain (GCF_000970345.1) contained 5,510 genes.

The genome sequence drafts presented here (BRIP 72872a and BRIP 72957a) expand the geographical range of available *R. andropogonis* genomes.

### Data availability.

The data from this project have been deposited under NCBI BioProject PRJNA753628. The raw sequencing data have been sent to the Sequence Read Archive under accessions (BRIP 72957a, SRR15602133; BRIP 72872a, SRR15602134) and the draft genome assembly has been sent to GenBank (BRIP 72872a, SAMN20702924; BRIP 72957a, SAMN20702925). The versions described in this paper are the first versions (SAMN20702924 and SAMN20702925). The annotated genomes have been released in the NCBI nucleotide database under the following accessions: JAIFTJ000000000 for BRIP 72872a and JAIFTI000000000 for BRIP 72957a.
